# Routine health information utilization and associated factors among health care professionals working at public health institution in North Gondar, Northwest Ethiopia

**DOI:** 10.1186/s12913-018-3498-7

**Published:** 2018-09-04

**Authors:** Eshetu Dagnew, Solomon Assefa Woreta, Atsede Mazengia Shiferaw

**Affiliations:** 1 Gondar specialized hospital, Gondar, Ethiopia; 2 Department of Health informatics, University of Gondar, P.o.box: 196, Gondar, Ethiopia

**Keywords:** Routine health information utilization, Health care professionals, North Gondar, Ethiopia

## Abstract

**Background:**

Routine health information systems (RHIS) are vital for the acquisition of data for health sector planning, monitoring, and evaluation. However, in developing countries the insufficient quality of the data produced by RHIS limits their usefulness in decision-making. As routine health information utilization is still low in Ethiopia, this study aimed to assess the magnitude of routine health data utilization and associated factors among health care professionals in some public health institutions in North Gondar, northwest Ethiopia.

**Methods:**

An institution based cross-sectional study was conducted from March to April2017, at public health institutions of North Gondar Zone, northwest Ethiopia. A total of 720 health care professionals were selected from public health institutions using the multi-stage sampling technique. Data were collected using a structured self-administered questionnaire and an observational checklist, cleaned, coded, and entered into Epi-info version 3.5.3 and transferred into SPSS version 20 for further statistical analysis. In the multiple logistic regression analysis, a less than 0.05 P-vale was considered statistically significant.

**Result:**

In this study, the level of good routine health information utilization among health professionals was 78.5% (95% CI: 73.2%, 84.3%). According to the multivariable logistic regression analysis, sex (AOR = 2.19, 95% CI: 1.47, 3.27), type of institution (AOR = 3.57, 95% CI: 2.39, 5.32), standard indicators (AOR = 3.28, 95% CI: 1.90, 5.65), data analysis skills (AOR = 1.90, 95% CI: 1.12, 3.23), and good governance (AOR = 1.97, 95% CI: 1.31, 2.95), were found significantly associated with a good level of health information utilization.

**Conclusion:**

Over three-fourths of the health care professionals working at public health institutions of North Gondar utilized health information better than the respondents in previous studies. Sex, type of institution, standard indicators, data analysis skills, and governance were factors related to routine health information utilization. Therefore, standard indicators, data analysis skills and good governance are highly recommended for improving routine health data utilization of health care professionals working at public health institutions.

**Electronic supplementary material:**

The online version of this article (10.1186/s12913-018-3498-7) contains supplementary material, which is available to authorized users.

## Background

Health information system (HIS) is a system designed for the collection, processing, use, and dissemination of health related data to improve health care outcomes. It is one of the six fundamental blocks of a health care system which includes health information system resources, indicators, data sources, data management, information products, dissemination, and use [1–3]. HIS involves the essentials for the overall health system which informs decision making in each of the other five blocks of the system and important in improving clinical and managerial decisions for providing quality information for evidence-based health practices [4, 5]. Data generated from healthcare facilities at regular intervals (routine health information system) are vital not only for the planning, monitoring, and evaluation of health care service activities, but also for the day-to-day patient management, health education, resource allocation, disease prioritization, and decision making [1, 6]. A properly functioning routine HIS gets the right information into the right hands at the right time, enabling policymakers, managers, and service providers to make decisions based on evidence, ultimately leading to sustainable health outcomes in the community they serve [1, 7, 8].

Globally, significant human and financial resources have been invested to improve routine health information systems for planning, reporting, community health mobilization, and observing disease trends. Recently, more attention has been given to strengthen evidence-based decision through good governance, transparency, and accountability. However, health system managers in developing countries tend to miss the role of routine data in tracking the performance of health programs and the overall health system, and neglect their typically used part of the performance of evaluation of district priority health targets [3, 8–10]. As a result, many health systems fail to fully link evidence to decisions and suffer from a reduced ability to respond to priority health needs at all levels of the health care system [1, 11]. Health information systems in low and middle income countries face challenges of inadequate data analysis; as a result the utilization of routine data for decision making remains very weak [12–14]. Too often, data are sat in reports, shelves, cabinets, databases and left unanalyzed to be sufficiently utilized for policy and program improvements [15, 16].

Routine health information system is vital for operational, tactical, and strategic decision making. Utilization of information at all levels of the health system through effective data analysis, interpretation, and utilization is important. However, poor data quality (incompleteness and incorrectness), and limited use remain the major concerns [3, 17]. Most health care providers in developing countries simply report routine health data without adequate utilization and feedback, and health care providers and managers at lower levels of the health care system have minimum understanding of the benefits of information. Findings from Africa indicate that routine health information utilization remains low [18, 19], 42% in Tanzania, 59% in Uganda [20], 58% in Liberia, and 65% of the health workers in South Africa [21] use routine health data for planning, management of health commodities, detecting outbreaks, and monitoring the performance of the health system. Despite efforts made to improve Health Management and Information System, data quality and use remain inadequate at peripheral health systems which are responsible for health care delivery [17]. Studies at various places in Ethiopia reported that routine health information utilization ranged from 22.5–69% [2, 22–29].

Reports showed that routine health information utilization can be affected by organizational [1, 30], technical, and behavioral characteristics of health care professionals [1]. Among the factors reported, analysis skills [24, 31, 32], lack of culture of information use [32], lack of supervision and regular feedback [22, 24], organizational infrastructure and HMIS training [24], knowledge, work load, computer skill, computer access, availability of HMIS guidelines and formats [29], availability of human resources [33], and data quality [15] are commonly associated with routine health information utilization. A report from Uganda showed that health care workers who lack training on computer software, data management, and HMIS were unable to understand the standard indicators and quality of data, subsequently making a limited use of routine health data [34, 35].

The Federal Ministry of Health (FMoH), Ethiopia introduced an Information Revolution to strengthen the method and practice of collecting, analyzing, and disseminating information for decisions. The revolution was targeted not only at changing the techniques of data and information management but also at bringing fundamental cultural and attitude change regarding the practice of information utilization. More of the strategy promoted the utilization data generated from peripheral health care systems. However, district facility staff rarely used routine data to identify performance gaps, make plans, and monitor progress. Information used health data only for report purposes and not to drive decisions and program improvements [35, 36]. Furthermore, the inadequacy of information use among health care professionals at the study setting remains a problem. Therefore, this study aimed to assess routine health information utilization among health care professionals and its predictors in North Gondar government health institutions. The finding will help to effectively implement different health sector programs and strategies, including the Health Sector Development Program (HSDP), social and community health insurances, and health care financing to address constraints in performance fulfillment of targets at district health care level.

## Methods

### Study design and setting

An institution-based cross-sectional study was conducted at public health institutions of North Gondar Administrative Zone from March to April 2017. The city of Gondar is located 747 km from Addis Ababa, the capital of Ethiopia. According to the plan and program report of the administrative zone health department, there are 2244 heath care professionals working at hospitals and health centers.

### Study participants, sample size, and sampling procedure

All health care professionals in selected health facilities were included in the study. Sample size was calculated using the single population proportion formula, assuming 53.3% prevalence of health information utilization in eastern Ethiopia [23], a 95% level of confidence, a 5% of margin of error, a design effect of 2, and a 5% of non-response rate. Finally, a minimum sample of 720 was obtained. In the zone, there were a total of nine hospitals and 135 health centers. Out of the total health facilities, 3 hospitals and 45 health centers were selected by the random sampling technique. Using proportional allocation technique, 720 health care professionals were selected.

### Data collection tool and procedure

The questionnaire was adapted from the PRISM frame work in which behavior, technical and organizational factors were the major determinants of the utilization of routine health information systems. Knowledge, data analysis skills, attitude, and the motivation of people who collect and use health information are behavioral factors, while data collection processes, systems, forms, and methods constitute the technical factors and organizational/environmental determinants, like information culture, structure, resources, governance, roles, and responsibilities of the health system affect the utilization of routine health information systems.

Data were collected using the pretested, structured, and self-administered questionnaire and an observational checklist. A two-days training was given to nine diploma graduate nurse data collectors and three BSc graduate nurse supervisors on the objective of the study and the confidentiality of information. During the course of data collection, participants were informed about the objective and processes of the study and confidentiality of the information. Data collectors were supervised at the study site and meetings were held with research assistants at the end of every day to discuss challenges and crosscheck data completeness and accuracy Additional file 1.

### Operational definition and study variables

The dependent variable, routine health information utilization, was measured by the PRISM conceptual framework on the system. It was defined as the use of routine health information for treating patients, disease prioritization, drug procurement, the day-to-day monitoring of health service activities, checking data quality, resource allocation, planning, department performance evaluation, evaluation of staff performance, selection of best experience within the health facility, sharing of health data to other facilities and stakeholders, decision making, and community mobilization and discussion. All these components of the assessment tool have likert scale measures, ranging from “strongly disagree” to “strongly agree”, finally, health workers’ mean scores were used to label health professionals’ health information utilization as “has good routine health information utilization” when they scored above the mean value, or “has poor routine health information utilization” when they scored equal to and below the mean value. Health care professionals in this study were defined as any health personnel who were collecting health data in order to utilize the information for the improvement of health status.

### Data processing and analysis

Data were entered into Epi-info version 7 and exported to the Statistical Package for Social Sciences (SPSS) version 20 for further analysis. Descriptive statistics, including frequencies and proportions, were computed using the binary logistic regression model in order to summarize variables. Variables with a *p*-value of less than 0.2 in the bi-variable analysis were entered into the multivariable logistic regression analysis. Both Crude Odds (COR) and Adjusted Odds Ratios (AOR) with 95% confidence interval were estimated to show the strength of associations. Finally, a *p*-value of less than 0.05 in the multivariable logistic regression analysis was used to identify variables significantly associated with the utilization of routine health information.

## Results

### Socio-demographic and behavioral characteristics

A total of 720 healthcare professionals were included in the study, giving response rate 100%. Just over half (52.1%) of the respondents were male health care professionals, 64.2% of whom were diploma graduate nurses. Slightly more than half (50.4%) nurses took part in this study than other health workers. Almost half (49.7%) of the respondents earned monthly salary of 100–150 dollar. More than half (58.6%) had positive belief in routine health information utilization. The majority (84.1%) gave no value to routine health information utilization, and 76.9% had no custom of routine health information utilization (Tables 1 and 2).

**Table 1:** Socio-demographic and economic characteristics of health care professionals working at public health institutions in North Gondar zone, Ethiopia, 2017

Variables	Frequency (*n* = 720)	Percentage (%)
Sex		
Male	375	52.1
Female	345	47.9
Level of education		
Diploma	462	64.2
BSc	249	34.6
Masters	9	1.3
Profession		
Medical Doctor	21	2.9
Health officer	56	7.8
Nurse	363	50.4
Midwife	114	15.8
Pharmacy	68	9.4
Laboratory	54	7.5
Environmental health	9	1.3
Occupational health	1	0.1
Nutrition	2	0.3
Psychiatry	3	0.4
Health informatics	19	2.6
Other health profession	10	1.4
Institution		
Hospital	223	31
Health center	497	69
Unit/Department		
Antenatal care (ANC)	37	5.1
Antiretroviral treatment (ART)	12	1.7
clinic	21	2.9
Delivery room	45	6.3
Drug dispensary Pharmacy	18	2.5
Emergency OPD	11	1.5
Expanded Program for	8	1.1
Immunization (EPI)	66	9.2
Family planning	23	3.2
Ward	12	1.7
HMIS office	51	7.1
Hygiene & sanitation	333	46.3
Laboratory	37	5.1
Outpatient Department (OPD)	15	2.1
Maternal and Child Health	20	2.8
(MCH)	11	1.5
Position		
OPD case team leader	65	9
Pharmacy head	5	0.7
Health center manager	19	2.6
Ward head	3	0.4
HMIS officer	19	2.6
Laboratory team leader	2	0.3
Staff	607	84.3
Salary (in dollar)		
50–100	181	25.1
101–150	356	49.7
151–200	84	11.7
201–250	57	7.9
251–300	32	4.4
> 301	8	1.1

**Table 2:** Behavioral characteristics of health care professionals working at public health institution in North Gondar zone, Ethiopia, 2017

Variable	Level of routine health information utilization (*n* = 720)	
	Good Frequency (%)	Poor Frequency (%)
Beliefs for routine health information utilization		
Positive	337 (79.9%)	85 (20.1%)
Negative	228 (76.5%)	70 (23.5%)
Custom for routine health information utilization		
Good	135 (81.3%)	31 (18.7%)
Poor	430 (77.6%)	124 (22.4%)
Value for routine health information utilization		
Good	90 (78.3%)	25 (21.7%)
Poor	475 (78.5%)	130 (21.5%)

### Organizational and technical factors

Of the total participants, 52, 57.6, 50.7 and 57% of had no culture of information utilization, had supervision on routine health information utilization (RHIU), had governance for RHIU and had good governance for RHIU. In addition, slightly higher than half (50.9 and 53.8%, respectively) used routine health data for planning, and received feedback on routine health information utilization. The majority (87.9% and 99.8%, respectively) of respondents received no training and no professional skill on RHIU. More than two-thirds (75% and 70.1%, respectively) had no professional knowledge about national indicators, and had no professional data analysis skills (Tables 3 and 4).

**Table 3:** Organizational characteristics of public health institutions in North Gondar zone, Ethiopia, 2017

Variable	Level of routine health information utilization (*n* = 720)	
	Good Frequency (%)	Poor Frequency (%)
Information use culture		
Yes	282 (81.5%)	64 (18.5%)
No	283 (75.7%)	91 (24.3%)
Supervision		
Yes	346 (83.4%)	69 (16.6%)
No	219 (71.8%)	86 (28.2%)
Governance		
Yes	348 (84.7%)	63 (15.3%)
No	217 (70.2%)	92 (29.8%)
Planning		
Yes	311(84.7%)	56 (15.3%)
No	254 (72%)	99 (28%)
Feedback		
Yes	324 (83.5%)	64 (16.5%)
No	241(72.6%)	91(27.4%)

**Table 4:** Technical characteristics of health care professionals at public health institutions in North Gondar zone, Ethiopia, 2017

	Type of training in the last 12 month	
	Trained	Untrained
Data collection	172 (24%)	548 (76%)
Data analysis	172 (24%)	548 (76%)
Information presentation	172 (24%)	548 (76%)
Information use	172 (24%)	548 (76%)

### Routine health information system utilization

In this study, the majority (94%) of the respondents used routine health data for treating patients, 90.1% for disease prioritization, 85% for drug procurement, 89.6% for monitoring day to day health service activities, 92.6% for checking data quality, 86.7% for resource allocation, 89% for planning, 88% for department performance evaluation, 86.5% for evaluation of staff performance, 85% for selection of best experience within a health facility, 82.8% for sharing health data to other facilities and stakeholders, 87.8% for decision making, and 87.1% for community mobilization and discussion. Good routine health information system utilization was noted among 78.5% of the health care professionals. The proportion of good health information utilization was 84.9% at health centers and 64.1% at hospitals Table 5.

**Table 5:** Routine health information utilization of health care professionals at public health institutions in North Gondar zone, Ethiopia, 2017

Activities used from routine health information utilization	(*n* = %565)	
	Yes	No
For treating patient	531(96%)	34(6%)
For disease prioritization	509(90.1%)	54(9.9%)
For drug procurement	482(85.3%)	83(14.7%)
For monitoring day to day health service activities	506(89.6%)	59(10.4%)
For checking data quality	523(92.6%)	42(%)
For resource allocation	490(86.7%)	75(13.3%)
For departments performance evaluation	497(88%)	68(12%)
For planning	503(89%)	62(11%)
For monitoring the performance of staffs	489(86.5%)	76(13.5%)
For selecting good experience with in the facility	480(85%)	85(15%)
For sharing of best experience for other facility and stakeholders	468(82.8%)	97(17.2%)
For decision making	496(87.8%)	69(12.2%)
For community mobilization and discussion	492(87.1%)	73(12.9%)

### Factors associated with good routine health information system utilization

In the bivariable logistic regression analysis, sex, type of facility, standard indicator, data analysis skills, culture of information, supervision, governance, planning and position were factors associated with good routine health information utilization at a *p*-value of less than 0.2. Consequently, these variables were subjected to multivariable logistic regression analysis, and it was noted that sex, type of facility, standard indicators, data analysis skills and governance were significantly associated with good routine health information utilization at a *p*-value of 0.05.

In this study, the high odds of good routine health information system utilization were noted among male health care professionals [AOR = 2.91; 95% CI: 1.47, 3.27], types of facility [AOR = 3.57; 95% CI: 2.39,5.32], standard indicators [AOR = 3.28; 95% CI: 1.90,5.65],, data analysis skills [AOR = 1.91; 95% CI: 1.12,3.23], and good governance [AOR = 1.91; 95% CI: 1.12,3.23] (Table 6).

**Table 6:** Factors associated with routine health information among health care professionals at public health institution in North Gondar zone, Ethiopia, 2017

	Routine health information utilization (*n* = 720)		COR (95% CI)	AOR (95% CI)
	Poor	Good		
Sex				
Male	67 (17.87%)	308 (82.13%)	1.55 (1.08, 2.22)	2.19 (1.47,3.27)^**^
Female	87 (25.22%)	258 (74.78%)	1	1
Type of institution				
Hospital	80 (35.87%)	143 (64.13%)	1	1
Health center	74 (14.89%)	423 (85.11%)	3.19 (2.21,4.62)	3.57(2.39,5.32)^**^
Professionals skill				
No	149 (22.51%)	513 (77.49%)	1	
Yes	5 (8.62%)	53 (91.38%)	3.08 (1.21,7.84)	
Standard indicator				
No	134 (28.63%)	334 (71.37%)	1	1
Yes	20 (7.94%)	232 (92.06%)	4.65 (2.83,7.66)	3.28 (1.90,5.65)
Data analysis skills				
No	130 (25.74%)	375 (74.26%)	1	1
Yes	24 (11.16%)	191 (88.84%)	2.76 (1.73,4.41)	1.91(1.12,3.23)^**^
Information use culture				
Poor	91 (24.33%)	283 (75.67%)	1	
Good	63 (18.21%)	283 (81.79%)	1.44 (1.01,2.07)	
Supervision				
No	85 (27.87%)	220 (72.13%)	1	
Yes	69 (16.63%)	346 (83.37%)	1.94(1.35,2.77)	
Governance				
Poor	92 (29.77%)	217 (70.23%)	1	1
Good	62 (15.09%)	349 (84.91%)	2.38 (1.65,3.43)	1.96 (1.31,2.95)^**^
Planning				
No	98 (27.76%)	255 (72.24%)	1	
Yes	56 (15.26%)	311 (84.74%)	2.13 (1.47, 3.08)	
Feedback				
No	90 (27.11%)	242 (72.89%)	1	
Yes	64 (16.49%)	324 (83.51%)	1.88 (1.32, 2.70)	
Position				
Management member	28 (29.79%)	66 (70.21%)	1	
Staff	126 (20.13%)	500 (79.87%)	1.68 (1.04, 2.73)	

^**^Variable significant at *p*-value less than 0.05

## Discussion

The aim of this study was to identify factors associated with poor routine health information utilization of health care professionals at public health institutions. According to our findings, the magnitude of routine health information utilization of health care professionals was 78.5%. This finding is higher than that of a study conducted in North Gondar (22.5%) [22], Jimma (32.9%) [23], East Gojjam (45.8%) [24], Hadiya zone (69%) [25], west Amhara (38%) [29], and in Ethiopia (48%) [2]. This variation might be due to differences in study periods. Besides, recently the government has given a special emphasis to utilization of information for evidence based decision making and the improvement of health care professionals’ information using culture [8]. Similarly, the finding was higher than those of studies reported from outside Ethiopia, that is Uganda (59%) [20], South Africa (65%) [21]. This might be due to variations in study periods and the criteria for measuring routine health information use [15].

According to the multivariable logistic regression analysis, the higher odds of routine health information utilization were noted among health professionals who had good governance when compared to health care professionals who had poor governance. The finding was supported by those of other studies reported elsewhere [1]. This might be due to the fact that the presence of good governance at public health institutions encourages staff to use routine health information for evidence based decision by strengthening health information systems [2]. Routine health information utilization among male respondents was higher than among females. It was revealed that the odds of health information utilization among male health professionals were 2.9 times higher than among females. This might be due to the fact that the majority of respondents were male.

Among the reported significant organizational factors, higher odds of routine health information utilization were noted among health professional working at health centers compared with those working at hospitals. This finding was supported by that of a study conducted in East Gojjam zone [24]. This might be due to the attention given by the government to district health facilities in terms of supervision and regular feedback [28, 37].

In our work, the odds of routine health information utilization of health care professionals who had data processing skills were 1.9 times more likely compared to health care professionals who had no data processing skills. This might be due to the fact that data analysis skills are one of the inputs for utilizing routine health information. Thus, without turning data into information, it is difficult to utilize routine health information for evidence based decision making. This finding is supported by that of a study conducted in East Gojjam zone [24], that is, perhaps because data analysis skills are very important for turning data into information.

Furthermore, the odds of health information utilization among health care professionals who had standard indicators in their offices were3.28 times higher than those who no such indicators to utilize routine health data. This might be due to the presence of data sources (standard indicator) which provides utilization of information for evidence based decision making [1].

## Conclusion

This study found that more than three fourths of the health care professionals at public health institutions in North Gondar had good routine health information utilization. Sex, standard indicators, good governance, and type of health facility had significant associations with routine health information utilization. Therefore, the standard indicators, and good governance at public health institutions are highly recommended. The study also suggested further investigations on the culture of health information utilization among health care providers where routine data are generated.

## Additional file

Additional file 1: Questionnaire to assess routine health information utilization and associated factors in north Gondar, northwest Ethiopia. (DOCX 36 kb)

## Abbreviations

AOR: Adjusted Odds Ratio
CI: Confidence Interval
RHIS: Routine Health Information System
SPSS: Statistical Package for Social Science
