# Supplementary material for: Routine health information utilization and associated factors among health care professionals working at public health institution in North Gondar, Northwest Ethiopia
Source: BMC Health Serv Res. 2018 Sep 4;18:685. doi: 10.1186/s12913-018-3498-7 (PMC6122568; doi:10.1186/s12913-018-3498-7)
Supplement: Supplementary file 1 — Questionnaire to assess routine health information utilization and associated factors in north Gondar, northwest Ethiopia. (DOCX 36 kb) [file 12913_2018_3498_MOESM1_ESM.docx]

Table: Questionnaire to assess routine health information utilization and associated factors

| **Section one: Socio demographic related equations.** | | | | | | | | | | |
| --- | --- | --- | --- | --- | --- | --- | --- | --- | --- | --- |
| 1. Age (in complete year) | | | | | | | | | | |
| 1. Sex Male Female | | | | | | | | | | |
| 1. Level of education Diploma BSc Master PhD | | | | | | | | | | |
| 1. Profession Medical Doctor Public Health officer Nurse Midwife Pharmacy Laboratory Environmental Health   Occupational health Nutrition  Health Informatics Other please specify | | | | | | | | | | |
| 1. Type of institution you working in Hospital Health Center | | | | | | | | | | |
| 1. Unit/Department currently you are working | | | | | | | | | | |
| 1. Current position in the organization | | | | | | | | | | |
| 1. Monthly salary (USD$)______________________ | | | | | | | | | | |
| **Section two: Technical Factors** | | | | | | | | | | |
| 1. Did you ever attend training on Health Information System (HIS) the last 12 month?   Yes No | | | | | | | | | | |
| 1. If your answer is yes for question number 9, in which topic you train?   **(You can choose more than one answer)**  Health information collection Health information presentation  Health information analysis Health information use | | | | | | | | | | |
| 1. Is there on job training program in your institution? Yes No | | | | | | | | | | |
| 1. If your answer is yes for question number 11, who provides the training?   **(You can choose more than one answer)**  RHIS focal person For data collectors For all health worker  Unit/Department head All interested staffs Other please specify | | | | | | | | | | |
| 1. Do you take orientation on routine health information utilization during your employment? Yes No | | | | | | | | | | |
| 1. Is there daily recording system for the activities? Yes No | | | | | | | | | | |
| 1. If the answer for question 14 is yes, who records the daily activities? By myself Other health professional Other please specify | | | | | | | | | | |
| 1. Have you use electronic device in routine health information utilization?   Yes No | | | | | | | | | | |
| 1. If your answer is yes for question number 16, for what purpose do you use?   **(You can choose more than one answer)**  Health information collection Health information report  Health information analysis Other please specify   1. Which information system is practiced on day-to-day work activity?   **(You can choose more than one question)**  Health information collection Health information presentation  Health information analysis Health information use | | | | | | | | | | |
| 1. Do you understand standard health indicators?   Yes No | | | | | | | | | | |
| 1. Do you have standardized set of indicators in your working office? | | | | | | | | | | |
| 1. In your institution, have you displayed health indicator targets?   Yes No | | | | | | | | | | |
| 1. Have you discuss the monthly performance progress using the standard indicators?   Yes No | | | | | | | | | | |
| 1. What are the common conventional tools used for data collection in your institution? **(You can choose more than one answer)**   Client cards Registration book  Tally sheets Other please specify | | | | | | | | | | |
| 1. Is the tool for data collection correctly and completely fill by the health professionals always? (check the answer by observation) Yes No | | | | | | | | | | |
| 1. Have you change the collected data into information in your department? (Check by observation) Yes No | | | | | | | | | | |
| 1. Have you reported the collected data in the last three months? (check the answer by observation) Yes No | | | | | | | | | | |
| **Section 3:Organizational factor** | | | | | | | | | | |
| 1. Does your facility system encouraging the culture of data use? Yes No | | | | | | | | | | |
| 1. Have you use routine health information for decision making in your organization?   Yes No | | | | | | | | | | |
| 1. Have you aware of your responsibilities in the organization? Yes No | | | | | | | | | | |
| 1. Have you seen any award in your organization as a motivation of good work?   Yes No | | | | | | | | | | |
| 1. Do you have role to make decision relating to your responsibility? Yes No | | | | | | | | | | |
| 1. Have you experience of accountability for poor performance in your organization? Yes No | | | | | | | | | | |
| 1. Which organizational factor influence information use in your institution? **(You can choose more than one answer)** 2. Organizational rules Yes No 3. Financial resources Yes No 4. Organizational practice Yes No 5. Inadequate human resource Yes No 6. Poor leadership Yes No | | | | | | | | | | |
| 1. For whom your data is reported? **(you can chose more than one choice)**   To department/unit head To woreda office  To higher management body of the institution To zonal office  To HMIS officer To Minister of health  To regional office | | | | | | | | | | |
| 1. In what form the generated report is distributed?   Paper form SMS (Short Messaging Service)  Email Other please specify | | | | | | | | | | |
| 1. Your facility has data quality check system? Yes No | | | | | | | | | | |
| 1. Does your facility have regular monitoring and evaluation supervision for health information system? Yes No | | | | | | | | | | |
| 1. If the answer for question 37 is yes, how many times your unit/department was supervised in the last 6 month?   Every month Once  Quarterly Other (Please specify) | | | | | | | | | | |
| 1. If the answer for question 37 is yes, who did the supervision? **( you can chose more than one)**   Ministry of health Woreda health office  Regional health office The facility administrator  Zonal health office Routine health information commute | | | | | | | | | | |
| 1. Is the facility have well streamlined health information policies? Yes No | | | | | | | | | | |
| 1. Is the facility has action plan for routine health information system? Yes No | | | | | | | | | | |
| 1. Does your organization have regular meeting to improve health information utilization?   Yes No | | | | | | | | | | |
| 1. Have you receive regular feedback on your report? Yes No | | | | | | | | | | |
| 1. If the answer for question 43 is yes, in what interval receive the feedback?   For every report Every six month  Quarterly Annually  Other please specify | | | | | | | | | | |
| **Section four: Behavioral factor** | | | | | | | | | | |
| Please express your level of agreement using the Likert scale; 1-Strongly Disagree, 2 Disagree, 3-Neither Agree or Disagree, 4-Agree 5-Strongly agree. | | | | | | | | | | |
| 1. Health care providers demand for information | 1 | 2 | | 3 | | 4 | | 5 | | |
| 1. Health professionals have poor attitude toward data collection | 1 | 2 | | 3 | | 4 | | 5 | | |
| 1. Routine health information system is useless | 1 | 2 | | 3 | | 4 | | 5 | | |
| 1. Collecting information adds no value for my activity | 1 | 2 | | 3 | | 4 | | 5 | | |
| 1. The collected data is not customized to patients treatment | 1 | 2 | | 3 | | 4 | | 5 | | |
| 1. Routine health information data is useful for monitoring facility performance | 1 | 2 | | 3 | | 4 | | 5 | | |
| 1. Health institution’s staff document their activities and keep records | 1 | 2 | | 3 | | 4 | | 5 | | |
| 1. Routine health information outputs give feel committed in improving health status of the target community | 1 | 2 | | 3 | | 4 | | 5 | | |
| 1. Understand and appreciate my role and responsibilities regarding to managed routine health information | 1 | 2 | | 3 | | 4 | | 5 | | |
| 1. Frequent use of routine health information data collection had benefit of patient as well as health facilities | 1 | 2 | | 3 | | 4 | | 5 | | |
| 1. Decisions based on evidence improve services delivery | 1 | 2 | | 3 | | 4 | | 5 | | |
| **Section five : Routine health information use** | | | | | | | | | | |
| Please indicate your level of agreement on the following statements regarding the utilization of routine health information. The statements are expressed using the Likert scale; 1-Strongly Disagree, 2 Disagree, 3-Neither Agree or Disagree, 4-Agree 5-Strongly agree. | | | | | | | | | | |
| **Routine health information used for :** | | | | | | | | | | |
| 1. Treating patient | | | 1 | | 2 | | 3 | | 4 | 5 |
| 2. Disease prioritization | | | 1 | | 2 | | 3 | | 4 | 5 |
| 3. Drug procurement | | | 1 | | 2 | | 3 | | 4 | 5 |
| 4. Monitoring day to day health service activities | | | 1 | | 2 | | 3 | | 4 | 5 |
| 5. Checking data quality | | | 1 | | 2 | | 3 | | 4 | 5 |
| 6. Resource allocation | | | 1 | | 2 | | 3 | | 4 | 5 |
| 7. Departments performance evaluation | | | 1 | | 2 | | 3 | | 4 | 5 |
| 8. Planning | | | 1 | | 2 | | 3 | | 4 | 5 |
| 9. Monitoring the performance of staffs | | | 1 | | 2 | | 3 | | 4 | 5 |
| 10. Selecting good experience with in the facility | | | 1 | | 2 | | 3 | | 4 | 5 |
| 11. Sharing of best experience for other facility and stakeholders | | | 1 | | 2 | | 3 | | 4 | 5 |
| 12. Decision making | | | 1 | | 2 | | 3 | | 4 | 5 |
| 13. Community mobilization and discussion | | | 1 | | 2 | | 3 | | 4 | 5 |

**Observation Checklist**

| Health Facility : |  |  |
| --- | --- | --- |
| Observer: | Date : |  |
| Items | YES | NO |
| Presence of health facility RHIS targets displayed |  |  |
| Presence of health facility indicator performance charts,  graphs and table displayed |  |  |
| Presence of staff meeting minutes reflecting reports, data  and feedback from health facility or district discussed |  |  |
| Presence of action work plan relating identified data gaps  and how they were addressed |  |  |
| Presence of RHIS training manual and guide |  |  |
| Presence of RHIS supervisory checklist |  |  |
| presence of RHIS supervisory report |  |  |
| presence of data quality assurance checklist |  |  |
